# Treatment of acute exacerbation of liver-cirrhosis-associated portal vein thrombosis with direct-acting oral anticoagulant, edoxaban, used as an initial treatment in the early postoperative period after abdominal surgery: a case report

**DOI:** 10.1186/s13256-020-02651-y

**Published:** 2021-02-10

**Authors:** Junya Toyoda, Daisuke Morioka, Nobutoshi Horii, Gakuryu Nakayama, Norio Oyama, Fumio Asano, Yusuke Izumisawa, Masaru Miura, Yoshiki Sato, Itaru Endo

**Affiliations:** 1 Department of Surgery, Yokohama Ekisaikai Hospital, 1-2 Yamadacho, Naka-ku, Yokohama, 231-0036 Japan; 2 Department of Gastroenterological Surgery, Graduate School of Medicine, Yokohama City University, Yokohama, Japan

**Keywords:** Liver cirrhosis, Portal vein thrombosis, Direct-acting oral anticoagulant, Edoxaban, Early postoperative period after major abdominal surgery

## Abstract

**Background:**

Cirrhosis-associated portal vein thrombosis (CA-PVT) has been reportedly observed in 5–30% of cirrhotic patients. Moreover, the acute exacerbation of CA-PVT is likely to occur after certain situations, such as a status after abdominal surgery. Safety and efficacy of the direct-acting oral anticoagulant (DOAC) used for cirrhotic patients have been being confirmed. However, use of the DOAC as an initial treatment for CA-PVT appears still challenging especially in the early postoperative period after major surgery in terms of unestablished efficacy and safety in such occasion.

**Case presentation:**

We herein report a case of the acute exacerbation of CA-PVT in the early postoperative period after abdominal surgery, which was successfully treated with DOAC, edoxaban used as an initial treatment. The patient was a 79-year-old Japanese male with alcoholic cirrhosis. The patient suffered choledocholithiasis and had a mural chronic CA-PVT extending from the superior mesenteric vein to the portal trunk. He underwent open cholecystectomy and choledochotomy. Early postoperative clinical course was uneventful except for abdominal distension due to ascites diagnosed on postoperative day (POD)7 when hospital discharge was planned. Contrast enhancement computed tomography (CE-CT) taken on POD 7 revealed the exacerbation of the CA-PVT. Despite recommendation for extension of hospital admission with low molecular weight heparin treatment, the patient strongly hoped to be discharged. Unwillingly, we selected DOAC, edoxaban, as an initial treatment, which was commenced the day after discharge (POD8). As a result, the remarkable improvement of the exacerbated CA-PVT was confirmed by the CE-CT taken on POD21. Any bleeding complications were not observed. Although a slight residue of the CA-PVT remains, the patient is currently doing well 4 years after surgery and is still receiving edoxaban. Any adverse effects of edoxaban have not been observed for 4 years.

**Conclusions:**

A case of successful treatment of the acute exacerbation of CA-PVT with edoxaban was reported. Moreover, edoxaban has been safely administered in a cirrhotic patient for 4 years. The findings obtained from the present case suggest that DOAC can be used as an initial treatment for CA-PVT even in early postoperative period after major abdominal surgery.

## Introduction

Albeit relatively rare, patients with liver cirrhosis are at risk of developing portal vein thrombosis (PVT) irrespective of acute or chronic [1–4]. According to the current treatment guideline, acute PVT or exacerbation of chronic PVT is treated with low-molecular-weight heparin (LMWH) and subsequent oral anticoagulation with warfarin [1–4]. On the other hand, the use of direct-acting oral anticoagulants (DOAC) in cirrhotic patients has been increasingly reported [3] and safety of DOAC in cirrhotic patients has been being confirmed [3, 4]. Currently, DOAC has become the first-line treatment for various venous thromboembolisms [5, 6]. Regarding cirrhosis-associated PVT (CA-PVT), safety and efficacy of DOAC used as an alternative to traditional anticoagulation were reported in several studies [3, 4, 6]. Furthermore, DOAC has begun to be used as initial treatment for CA-PVT [6] despite absence of randomized control trials (RCT) to investigate the efficacy and safety of DOAC compared to the traditional anticoagulation.

On the other hand, CA-PVT has been reported to be likely to exacerbate under several situations, including a status after abdominal surgery [7, 8]. In such situation, it is still considered that traditional anticoagulation should be used as an initial treatment [2–4, 6–8]. Especially in the early postoperative period after major abdominal surgery, the safety and efficacy of DOAC used as an initial treatment for CA-PVT have not been examined [2–4, 6–8].

We herein report a cirrhotic patient receiving major abdominal surgery who showed the postoperative acute exacerbation of CA-PVT successfully treated with DOAC, edoxaban. In addition, edoxaban has been safely administered during long-term period in 4 years without any adverse events, including minor and/or major bleeding episodes, to a cirrhotic patient in the present case.

## Case presentation

**Fig. 1:**
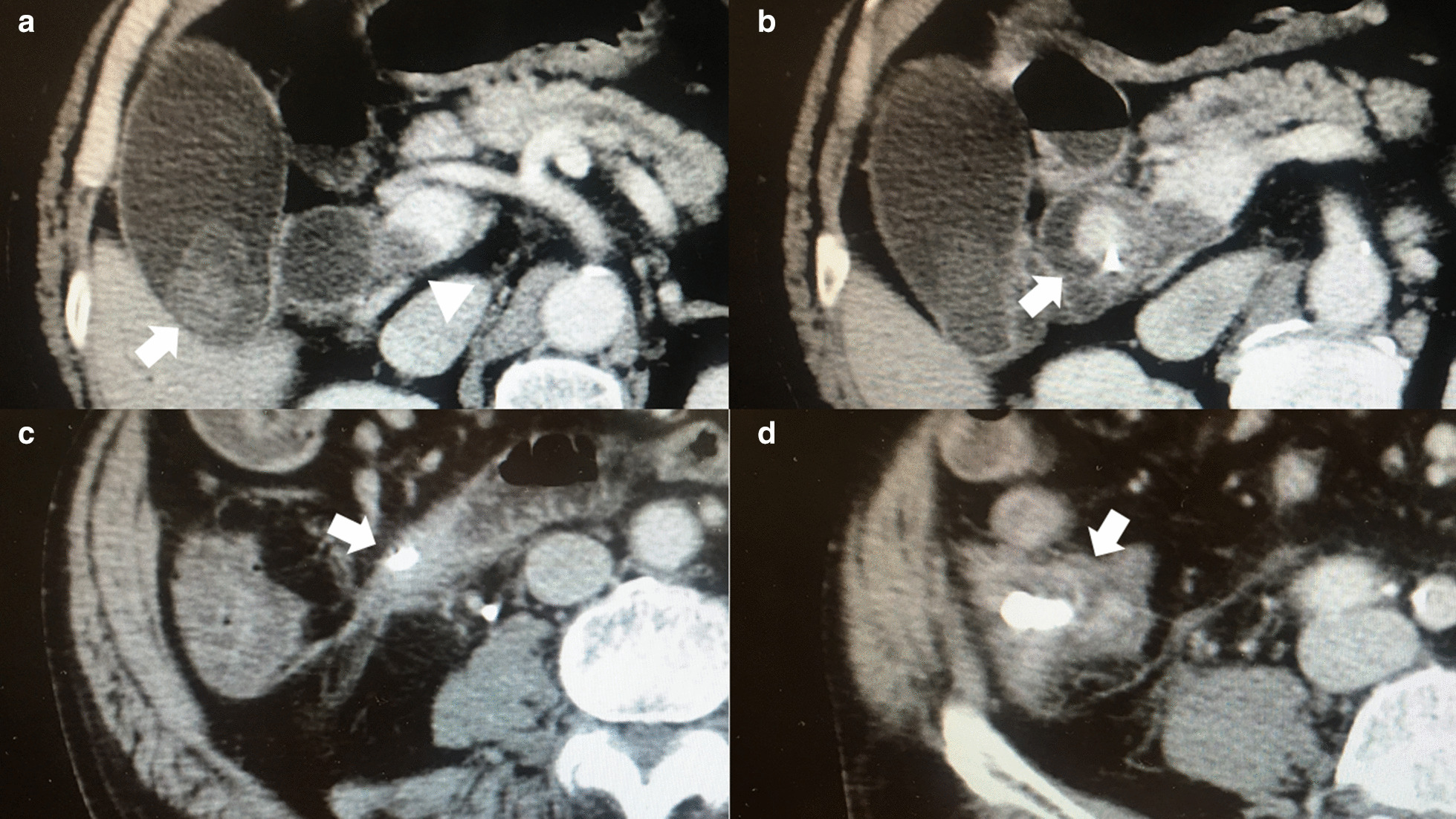
Findings of preoperative abdominal contrast-enhanced computed tomography regarding the choledocholithiasis and the endoscopically inserted biliary drainage tube. Several gallstones existed in the gallbladder and common bile duct (CBD). Maximum stone with 3cm in diameter was observed in CBD (White arrows). Furthermore, distal end of the endoscopically inserted biliary drainage tube penetrated through the duodenum to the cecum (White arrowheads). (*Ce* cecum; and *Du* duodenum)

**Fig. 2:**
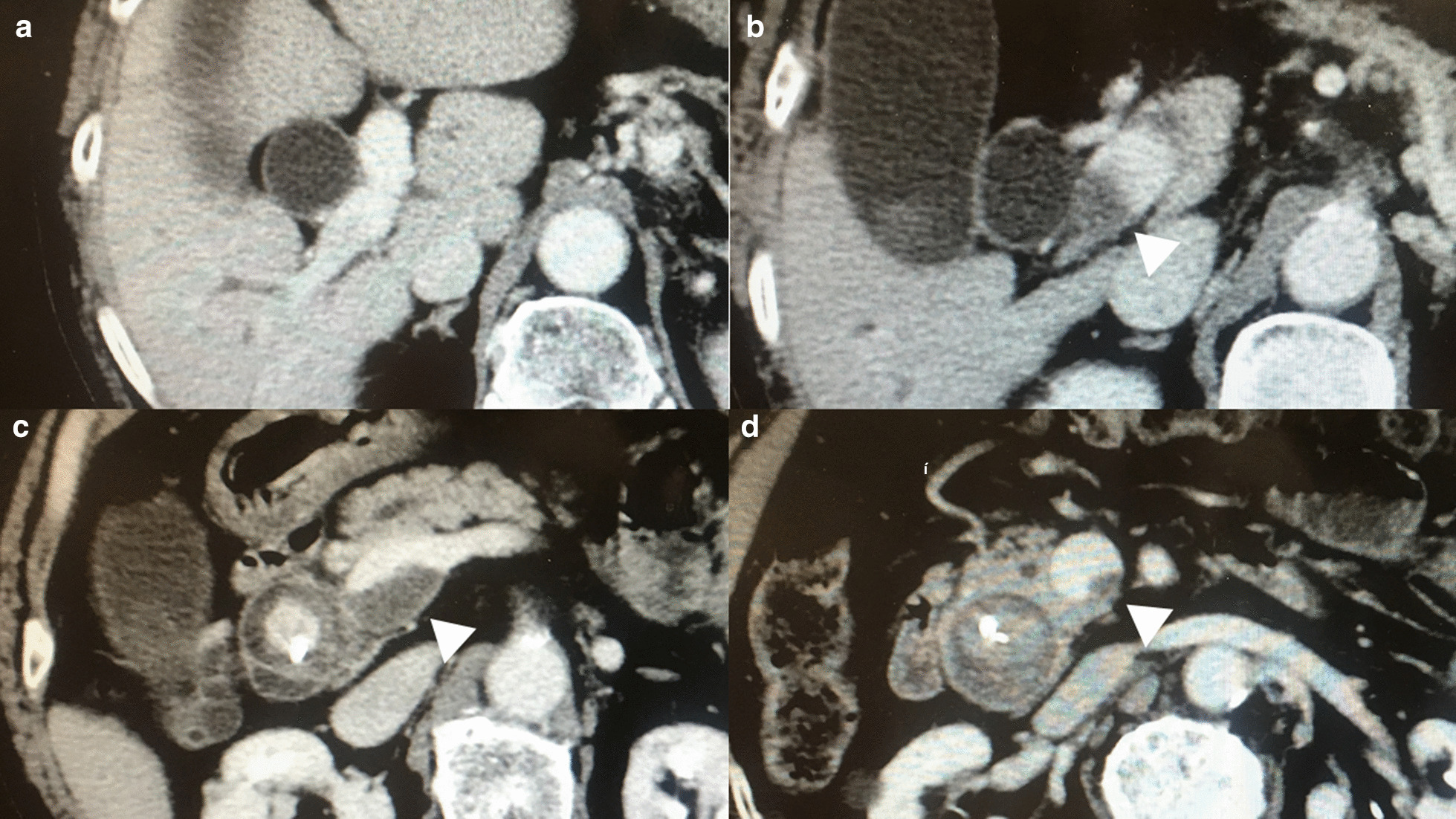
Findings of the portal vein thrombosis in the preoperative period. The portal vein thrombosis (PVT) (White arrowheads) extended from the superior mesenteric vein (**d**) to the portal trunk (**b**). Although the PVT caused considerable stenosis at the level of the confluence of the superior mesenteric vein and the splenic vein (**c**), it did not reach the bifurcation of the right and left portal veins (**a**)

The patient was a 79-year-old Japanese male with alcoholic liver cirrhosis who suffered 9-year history of choledocholithiasis (CL). He had neither any noticeable family history nor psychosocial history. Nine-year previously, the patient underwent endoscopic retrograde biliary drainage (ERBD) using plastic stent tube for CL. Surgery was recommended to the patient at that time. But the patient refused the recommendation. Thereafter, despite repeated episodes of cholangitis and/or abdominal pain due to failed ERBD, the patient refused the offer of not only surgery but also exchanging ERBD tube until when symptoms due to CL became unbearable and thus the patient hoped to receive surgery. Findings of preoperative abdominal contrast-enhancement computed tomography (CE-CT) were as follows. Several gallstones existed in the gallbladder and common bile duct (CBD). Maximum CBD stone was 3 cm in diameter. Furthermore, probably because ERBD stent tube was unexchanged more than 9 years, distal end of the ERBD tube penetrated through the duodenum to the cecum (Figure 1). In addition, a mural PVT extending from the superior mesenteric vein to the portal trunk was observed (Figure 2). However, patency of the portal venous systems was well maintained. Moreover, Child-Pugh classification was A and model for end-stage liver disease score was 9. Thus, the patient was considered endurable to major abdominal surgery. Therefore, open cholecystectomy and choledochotomy was performed. At the surgery, small cecostomy was opened and subsequently closed after removal of the ERBD stent tube through the cecostomy.

**Fig. 3:**
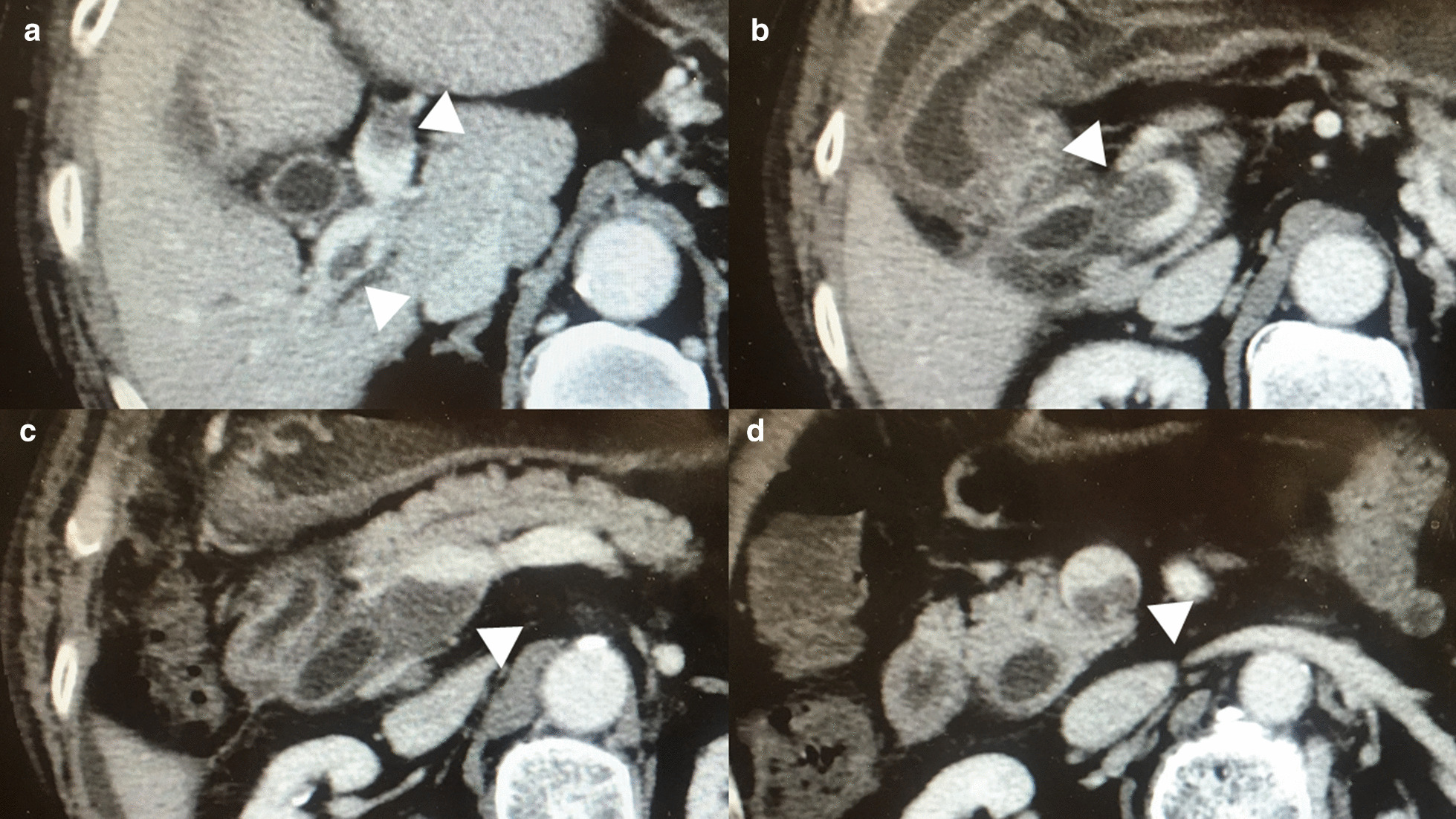
Findings of the portal vein thrombosis on postoperative day 8. Exacerbation of the portal vein thrombosis (PVT) (White arrowheads) was noticed with the findings of contrast-enhanced computed tomography taken on postoperative day 7. The PVT extended cephalically beyond the bifurcation of the right and left portal veins (**a**). The stenosis caused by the PVT was deteriorated either in the portal trunk (**b**) or in the confluence of the superior mesenteric vein and the splenic vein (**c**). Furthermore, the PVT extended much more caudally compared to the preoperative period (**d**). (*CHD* common hepatic duct; and *CBD* common bile duct)

**Fig. 4:**
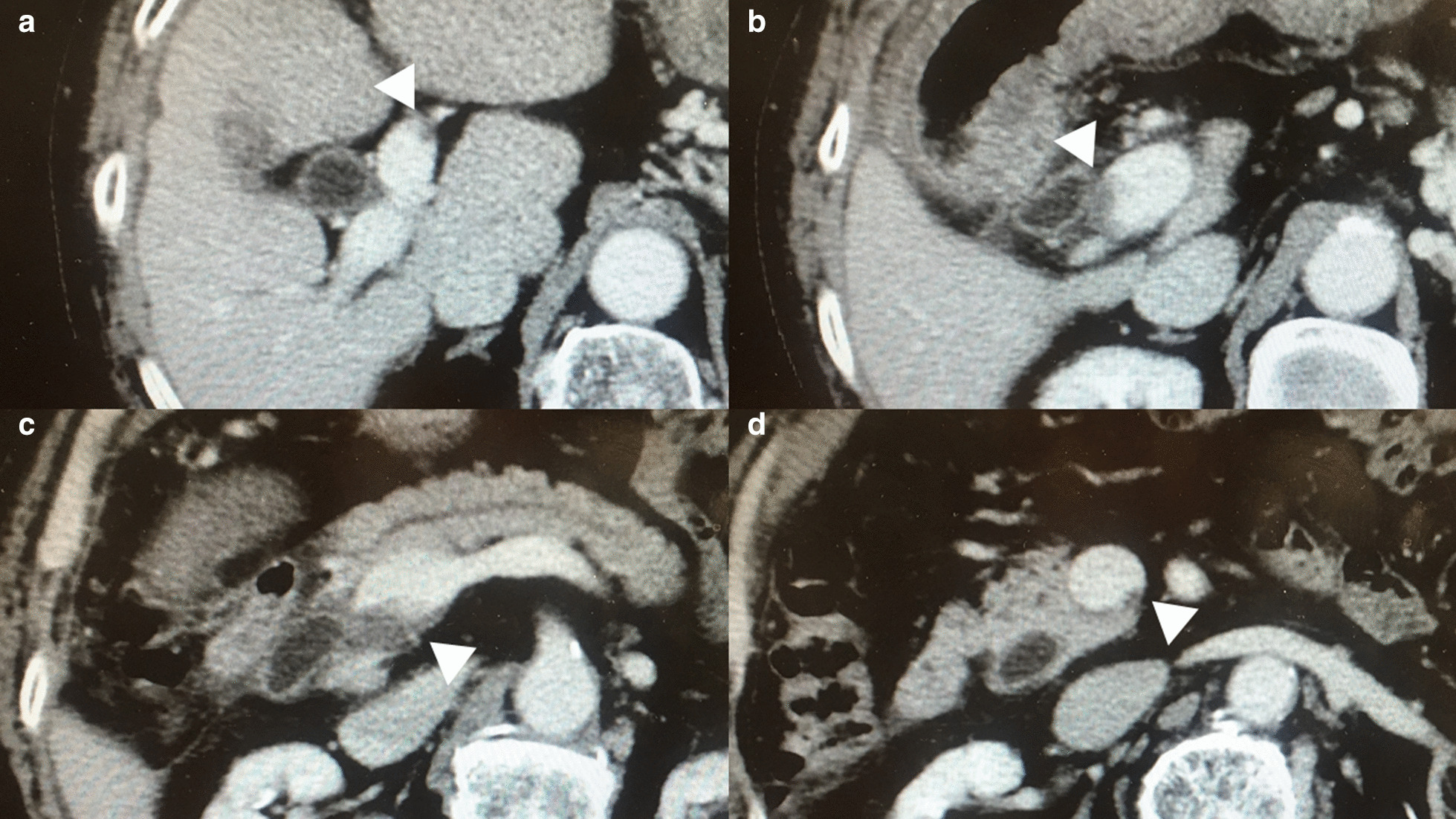
Findings of the portal vein thrombosis 20 days after the initiation of edoxaban. The portal vein thrombosis (PVT) (White arrowheads) was markedly shrunk. The cephalic end of the PVT was no longer observed in the right or left portal vein although very small residue was observed in the left portal vein (**a**). The stenosis caused by the PVT was markedly improved either in the portal trunk (**b**) or in the confluence of the superior mesenteric vein and the splenic vein (**c**). Furthermore, the caudal end of the PVT was markedly withdrawn cephalically compared to the prior evaluation (**d**). (*CHD* common hepatic duct; and *CBD* common bile duct)

Early postoperative course was uneventful until postoperative day (POD)7 when hospital discharge was planned. On POD7, however, the patient complained of abdominal distension due to moderate ascites, which was not observed in the preoperative period. CE-CT was taken on POD7 in search for the cause of the abrupt abdominal distention due to the ascites. The CE-CT revealed the exacerbation of the PVT. The distal end of the PVT was located markedly farer from the confluence of the superior mesenteric vein and the splenic vein. Moreover, the proximal end of the PVT was extended beyond the bifurcation of the right and left portal veins (Figure 3). Furthermore, the exacerbated PVT caused 90% stenosis of portal vein trunk, suggesting that the exacerbation of the PVT deteriorated portal hypertension, leading to the increased abdominal distension due to the development of ascites. Serum value of d-dimer at that time was elevated to 29.4 μg/mL on POD7 although the preoperative value was 3.3 μg/mL. We recommended the extension of hospital admission for receiving anticoagulant treatment with LMWH injection followed by oral warfarin, which was recommended as the first-line treatment in the current treatment guideline of PVT [1–4]. However, the patient strongly hoped to be discharged on POD7 because the patient felt that his general condition was well recovered except for abdominal distension. Thus, the patient was discharged from hospital on POD7. The day next to discharge (POD8), we initiated edoxaban therapy (30 mg once a day) after our institutional review board approved the use of DOAC as an initial treatment. Thereafter, the patient received weekly outpatient clinic follow-up. At the first outpatient visit after discharge (POD14), abdominal distension due to ascites was improved and his serum d-dimer dramatically decreased to 7.8 μg/mL. Twenty days after initiating edoxaban (POD28), serum d-dimer was recovered to 3.2 μg/mL, which was equivalent to the preoperative level. In addition, CE-CT taken on POD28 showed that PVT was markedly shrunk and patency of the portal vein trunk remarkably improved (Figure 4). In addition, ascites was eradicated. Thereafter, the patient received monthly outpatient clinic follow-up. Six months after surgery, the PVT was further improved. Because the PVT was not completely eradicated and mild elevation of his serum d-dimer sustained, edoxaban has been continued to date. However, the patient has been doing well 4 years after surgery without any adverse events of edoxaban, including minor and/or major bleeding complications.

## Discussion

Generally, the worse the cirrhosis, the more common the CA-PVT. Prevalence of the CA-PVT has been reported to range 5–30% of cirrhotic patients [1–4]. Furthermore, underlying CA-PVT has been reported to be easily exacerbated by various factors, including abdominal surgery [7, 8]. In the present case, although the PVT was considered as CA-PVT, inappropriate management of CL, in which ERBD tube stent was unexchanged during extremely long-term period with more than 9 years, might have had a negative impact on the underlying PVT.

Current treatment guidelines recommend LMWH and subsequent oral warfarin as the treatment of the CA-PVT [1–4]. However, LMWH needs once or twice daily subcutaneous injection [1–4]. Furthermore, warfarin necessitates frequent dose adjustments that often require several days. In addition, although warfarin works through inhibition of vitamin-K, the vitamin K-dependent coagulation factors are usually low in cirrhotic patients [9, 10]. In addition, LMWH does not work without antithrombin III, which is produced by the liver and thus usually lacking in cirrhotic patients [11].

On the other hand, DOAC was considered to be a new treatment option for venous thromboembolism (VTE) in patients with cirrhosis and has been increasingly used because superiority of DOAC to traditional anticoagulation includes quick onset of action, oral dosing, and that it does not necessitate routine monitoring for drug levels or effect although the safety and efficacy of DOAC for CA-PVT have not been confirmed in RCT [3, 4, 12].

In the present case, an underlying PVT was exacerbated in the early postoperative period. The exacerbation seemed quite severe because of prominent stenosis of portal trunk. Therefore, we considered that the situation must be urgently intervened and thus we recommended the extension of hospital admission for receiving LMWH therapy to the patient. However, the patient rejected the recommendation and strongly hoped to be discharged on the day (POD7) when the exacerbation of the PVT was proven. Although high level evidence for the safety and efficacy of DOAC for CA-PVT is lacking, recent studies showed that anticoagulation therapy with DOAC in cirrhotic patients did not differ in safety and efficacy compared to the traditional anticoagulation [4, 10]. Thus, we decided to use DOAC for the treatment with expectation of its prompt effect-expression. With regard to the reason why we selected edoxaban among DOACs, edoxaban is a coagulation factor Xa inhibitor and has been a sole DOAC approved for VTE prophylaxis after lower limb arthroplasty in the Japanese Social Insurance coverage [13] although any DOACs have not been approved for VTE prophylaxis after abdominal surgery in Japan. In other words, only edoxaban can be used in the early postoperative period after major surgery in Japan. In addition, preparative cessation of edoxaban for major surgical and/or endoscopic invasive procedures requires only one dose [14, 15] although other DOACs, including rivaroxaban (coagulation factor Xa inhibitor), apixaban (coagulation factor Xa inhibitor), and dabigatran (thrombin inhibitor), necessitate at least 2 days of cessation before major invasive procedures [16]. As to dabigatran, a reversal agent, idarucizumab, has been recently approved for acute hemorrhagic events and/or necessity of emergent invasive procedures but it is quite expensive [17]. In cirrhotic patients, the early postoperative bleeding complications, such as postoperative bleeding or gastrointestinal bleeding, may be likely to occur compared to non-cirrhotic patients. From this aspect, the fact that edoxaban necessitates only one dose preparative cessation for invasive procedures appears beneficial in the situation of the present case.

Some reports indicate usefulness of rivaroxaban for PVT in patients with cirrhosis [18–20]. However, cirrhotic patients with moderate or severe hepatic impairment (Child-Pugh classification B or C) showed increased pharmacodynamic effects of rivaroxaban, i.e. the risk of bleeding episodes is considered to be increased [21]. Liver function of the present case corresponded to Child-Pugh B in the early postoperative period even though Child-Pugh A in the preoperative period. Metabolism of edoxaban was reported to be less dependent on hepatic function compared to rivaroxaban [22] Moreover, it was reported that pharmacokinetics of edoxaban and its metabolite M4 in patients with hepatic impairment were not different compared to patients without hepatic impairment [22]. These findings appear to support that the usage of edoxaban for the present case was quite reasonable. In fact, edoxaban has been safely administered for 4 years without any adverse effects in the present case. This safe and long-term administration of edoxaban suggests that our choice of edoxaban was justifiable.

In the current Japanese Social Insurance coverage, only enoxaparin and fondaparinux can be used for VTE prophylaxis after abdominal surgery [23, 24]. Of note, enoxaparin was reported to be efficacious for preventing PVT developing after hepatic resection for liver cancers [23]. Reportedly, new onset PVT in cirrhotic patients and/or exacerbation of CA-PVT is likely to occur after abdominal surgery [7, 8, 23]. Thus, it can be said that we should have used VTE prophylaxis for the present case. If VTE prophylaxis would have been used, acute exacerbation of CA-PVT might have not developed. The findings obtained from the present case suggest that edoxaban can be safely used in the early postoperative period after abdominal surgery. Thus, it seems that the indication of edoxaban can be expanded for the purpose of VTE prophylaxis after abdominal surgery. This expansion may lead to prevention of new onset PVT and/or exacerbation of CA-PVT after abdominal surgery in cirrhotic patients.

## Conclusion

We reported a case of the acute exacerbation of CA-PVT in the early postoperative period after major abdominal surgery, which was successfully treated with edoxaban. The present case suggests that edoxaban can be an alternative option to traditional anticoagulation in such challenging situation.

## Data Availability

The datasets used and/or analyzed during the current study are available from the corresponding author on reasonable request.

## Abbreviations

PVT: Portal vein thrombosis
LMWH: Low-molecular-weight heparin
DOAC: Direct-acting oral anticoagulant
CA-PVT: Cirrhosis-associated portal vein thrombosis
RCT: Randomized control trial
CL: Choledocholithiasis
ERBD: Endoscopic retrograde biliary drainage
CECT: Contrast-enhancement computed tomography
CBD: Common bile duct
POD: Postoperative day
VTE: Venous thromboembolism
